# Preclinical Experience Using 4D Intracardiac Echocardiography to Guide Cardiac Electrophysiology Procedures

**DOI:** 10.1111/jce.16531

**Published:** 2024-12-30

**Authors:** Colin J. Blumenthal, Weihow Hsue, Tiffany Chen, David Zhang, Erez Brem, Fermin C. Garcia, David J. Callans, Francis E. Marchlinski, Pasquale Santangeli, Cory M. Tschabrunn

**Affiliations:** ^1^ Division of Cardiovascular Medicine, Cardiac Electrophysiology Section Hospital of the University of Pennsylvania Philadelphia Pennsylvania USA; ^2^ Department of Clinical Sciences, College of Veterinary Medicine Cornell University Ithaca New York USA; ^3^ Division of Cardiology University of Washington Seattle Washington USA; ^4^ Geisinger Commonwealth School of Medicine Scranton Pennsylvania USA; ^5^ Biosense Webster Irvine California USA; ^6^ Department of Cardiovascular Medicine Cleveland Clinic Cleveland Ohio USA

**Keywords:** 4D ICE, left atrial appendage occlusion, moderator band ablation, NuVision, papillary muscle ablation

## Abstract

**Introduction:**

Intracardiac echocardiography (ICE) is an essential imaging modality for electrophysiology procedures, allowing intraprocedural monitoring, real‐time catheter manipulation guidance, and visualization of complex anatomic structures. Four‐dimentional (4D) ICE is the next stage in the evolution of the technology, permitting 360° rotation of the imaging plane, simultaneous multiplanar imaging, and volumetric acquisition, similar to transesophageal echocardiography (TEE). In this study, we report our experience with a novel 4D ICE catheter (NuVision, Biosense Webster) in structural electrophysiology procedures and difficult ventricular ablations in a swine preclinical model.

**Methods:**

7 Yorkshire swine underwent 4D ICE (NuVision, Biosense Webster) imaging procedures and anatomical shells of the RV, LV, and LA were created on the CARTO mapping system. Ablation was performed on the RV moderator band and LV papillary muscles under imaging guidance with the 4D ICE catheter. Additional ICE images were obtained of the LAA to simulate placement of a left atrial appendage occlusion (LAAO) device. Triphenyl tetrazolium chloride was administered before euthanasia and hearts were harvested, fixed in formalin, and sectioned.

**Results:**

CARTOSOUND reconstruction was completed using the novel multiplane imaging software platform, allowing for creation of anatomy with minimal movement of the ICE catheter. Maps generated were similar to 3D reconstruction acquired in pre‐procedure CT. Ablation lesions were successfully delivered to the LV papillary muscles and RV moderator band with excellent correlation between gross pathology, electroanatomic mapping (EAM), and ICE images. 2D, multiplane, and 3D volumetric images were obtained of the LAA with minimal catheter movement to simulate use for an LAAO procedure.

**Discussion:**

Intracardiac ultrasound has become an essential tool in the electrophysiology lab, especially for visualization of intracardiac structures in real time. 4D ICE is the natural progression of this technology, adding features previously only seen on TEE probes. In this preclinical study, 4D ICE was used to create CARTOSOUND shells with less catheter manipulation, which could decease procedural times and potentially decrease complications related to frequent manipulation of the ICE catheter. It was also placed in the left atrium to acquire multiplane and 3D rendered volumes of the left atrial appendage (LAA) similar to what would be required for an LAA occlusion procedure. This could be used as an alternative to TEE in LAAO procedures, potentially improving procedural efficiency and negating the need for general anesthesia. Additionally, it was used for real‐time ablation guidance, specifically directly on the RV moderator band and LV papillary muscles. Multiplanar imaging allowed for more accurate catheter visualization and localization when targeting these complex 3D intracavitary structures.

**Conclusion:**

4D ICE is the next stage in evolution of an essential imaging modality for electrophysiology procedures. Integration within the electroanatomical mapping system software platform may provide additional value for guiding ablation of challenging intracavitary structures and is a novel feature of the NuVision catheter. Through promising, this technology is new and further clinical investigation will be required to determine the ideal applications for its use.

## 1 Introduction

Intracardiac echocardiography (ICE) has emerged as an essential imaging modality for transcatheter cardiac interventions, allowing intraprocedural monitoring, real‐time catheter manipulation guidance, and visualization of complex anatomic structures otherwise indiscernible on fluoroscopy [1, 2]. Its ability to provide real‐time images of catheter contact and identify arrhythmogenic substrates makes it a critical tool in ventricular arrhythmia (VA) ablation [3], and has been shown to improve catheter ablation outcomes in VA patients with or without structural heart disease [4]. In addition, ICE is developing interest as an alternative to transesophageal echocardiography (TEE) in guiding structural electrophysiology and interventional cardiology procedures (e.g., left atrial appendage occlusion [LAAO], transcatheter edge‐to‐edge repair [TEER], PFO closure, transcatheter valve replacement, etc.) [5].

Similar to TEE, ICE offers significantly reduced fluoroscopic time, but also provides additional advantages including reduced procedural time and faster room turnover [6]. Additionally, it can be performed by the electrophysiologist and is better tolerated for longer procedures, eliminating the need for a second operator and general anesthesia [5, 6]. However, one main limitation of ICE is the lack of meaningful three‐dimensional (3D) volumetric imaging, which is essential for structural cardiology procedures. Although the recent arrival of the AcuNav‐V 3D ICE catheter introduced viewing depth and visualization from multiple perspectives, its limited 22° by 90° sample volume pales in comparison to the wide 3D sectors afforded by 3D TEE [7, 8].

In this study, we demonstrate the use of a novel four‐dimensional (4D) ICE catheter (NuVision, Biosense Webster) in structural electrophysiology procedures and difficult ventricular ablations. The NuVision NAV is a 10 F single‐use ultrasound imaging catheter with a 4D ICE ultrasound transducer capable of real‐time cardiac imaging. The catheter includes a unique sensor which conveys 3D location information that is integrated with the CART 3 EP Navigation System (Biosense Webster), allowing for high‐quality multiplanar reconstruction with minimal catheter manipulation. Not only is this technology potentially advantageous when compared to TEE in LAAO procedures, it could also be useful in ventricular ablations that require interaction with complex 3D structures (e.g., papillary muscles, moderator band) where full anatomical appreciation is challenging on 2D ICE. In this study, we describe the use of 4D ICE in a swine model for visualization of the left atrial appendage (LAA) as well as other critical ventricular structures.

## 2 Methods

The research protocol was approved by the Institutional Animal Care and Use Committee of the University of Pennsylvania and conformed to the position of the American Heart Association on the Use of Research Animals. All experiments were performed at the University of Pennsylvania Translational Cardiac Electrophysiology Laboratory in Philadelphia, Pennsylvania. A total of 7 Yorkshire swine (male, 50–60 kg) underwent 4D ICE (NuVision, Biosense Webster) imaging procedures with imaging software integrated with the CARTO mapping system. After 12 h of fasting, sedation was initiated using 15–30 mg/kg of intramuscular ketamine and endotracheal intubation was performed. General anesthesia was maintained with isoflurane inhalation (1.5%–4%). Percutaneous femoral arterial and venous access was obtained under vascular ultrasound guidance. After vascular access, 150 μ/kg of unfractionated heparin was administered intravenously with additional maintenance boluses given to maintain an activated clotting time of 250–300 s.

Imaging with the NuVision NAV catheter was performed in the RA, RV, and LA. Anatomical shells of the RV, LV, and LA were created on the CARTO mapping system. Endocardial tracings were taken manually with CARTOSOUND for sound to fast anatomical mapping (“FAM”) as artificial intelligence‐based auto‐contouring algorithms were not available.

Ablation was performed on the RV moderator band (20–30 W, 60 s) and the LV papillary muscles (30–40 W, 60 s) with a 3.5 mm open irrigated catheter (ThermoCool Smart Touch Surround Flow; Biosense Webster) with a target contact force of 8–14 g. The Smart‐Ablate ablation generator (Biosense Webster) was used with two return patches in all experiments, placed on the same dorsal side of the animal. For each ablation application, 4D ICE was used to monitor the lesion in real‐time and facilitate direct visualization of the catheter tip during lesion delivery. Contact force, temperature, and impedance data were also continuously monitored during each ablation lesion. Additional ICE images were obtained of the LAA.

After the experiment was completed, 10 mL of triphenyl tetrazolium chloride was administered intravenously 30 min before euthanasia to differentiate metabolically active tissue from inactive tissue. Following euthanasia, the hearts were harvested and fixed in formalin for > 7 days. After tissue fixation, the hearts were sectioned in 2‐mm slices perpendicular to the intraventricular groove from the apex to base. Ablation lesion applications were identified on gross pathology and correlated to the location on the electroanatomical map.

## 3 Results

**Figure 1:**
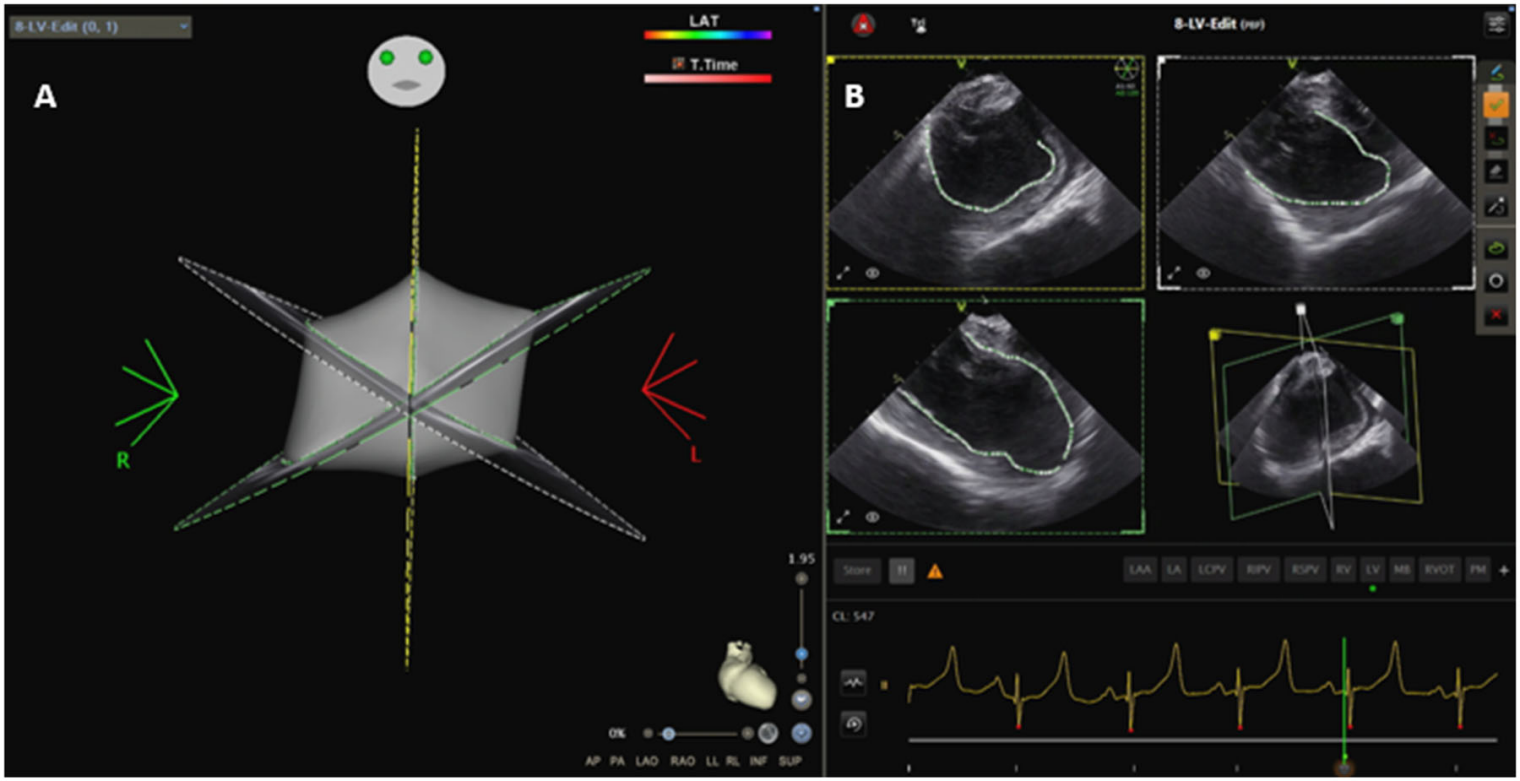
4D ICE creation of a left ventricular shell using CARTOSOUND and sound to FAM using multiplane acquisition (A). Ultrasound views of multiple simultaneously acquired planes allowing for contour creation in multiple planes without catheter manipulation (B).

**Figure 2:**
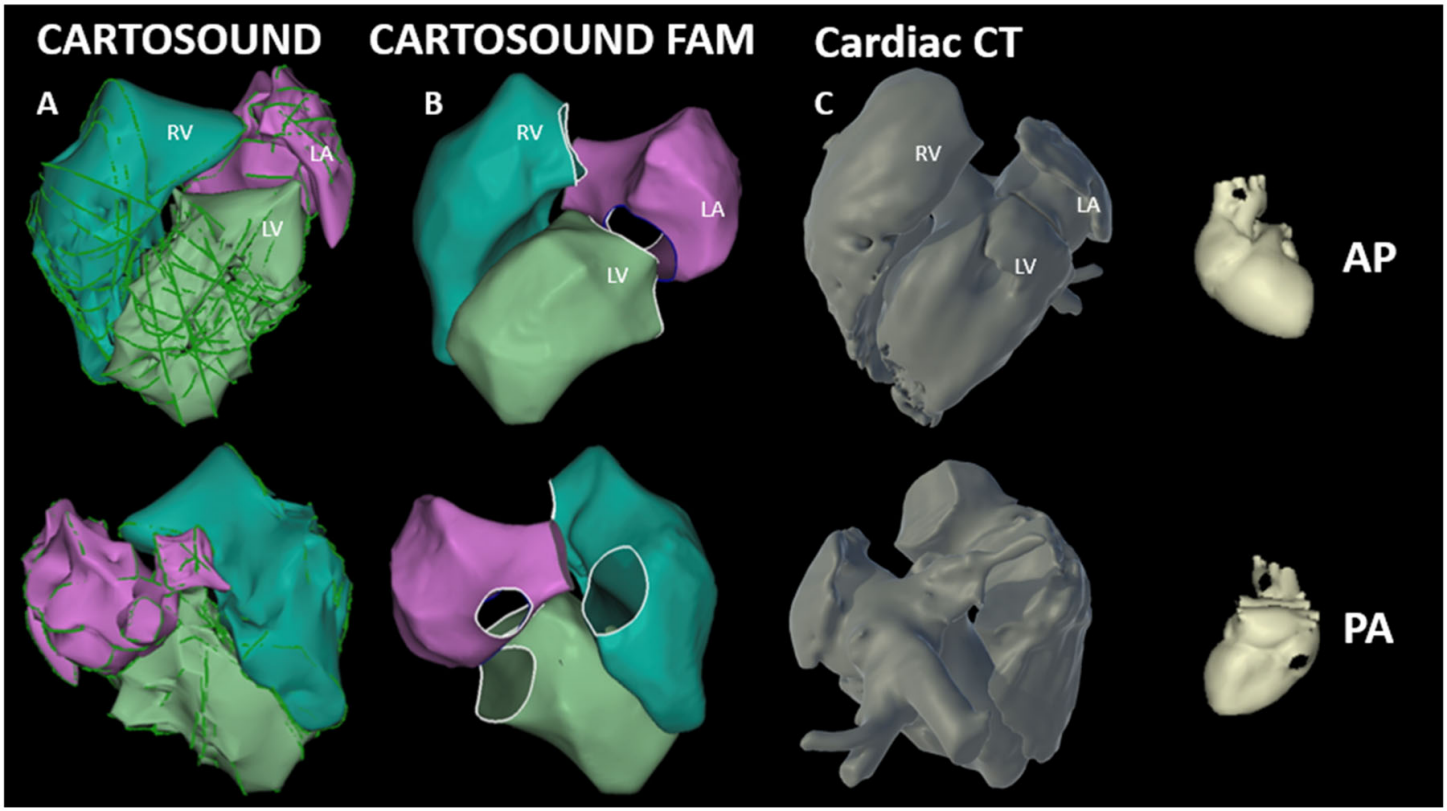
AP (top) and PA (bottom) images of a porcine RV (teal), LV (light green), and LA (pink). CARTOSOUND shell created with multiplane imaging with (A) and without (B) contours, compared to a 3D volume rendering by CT (C).

**Figure 3:**
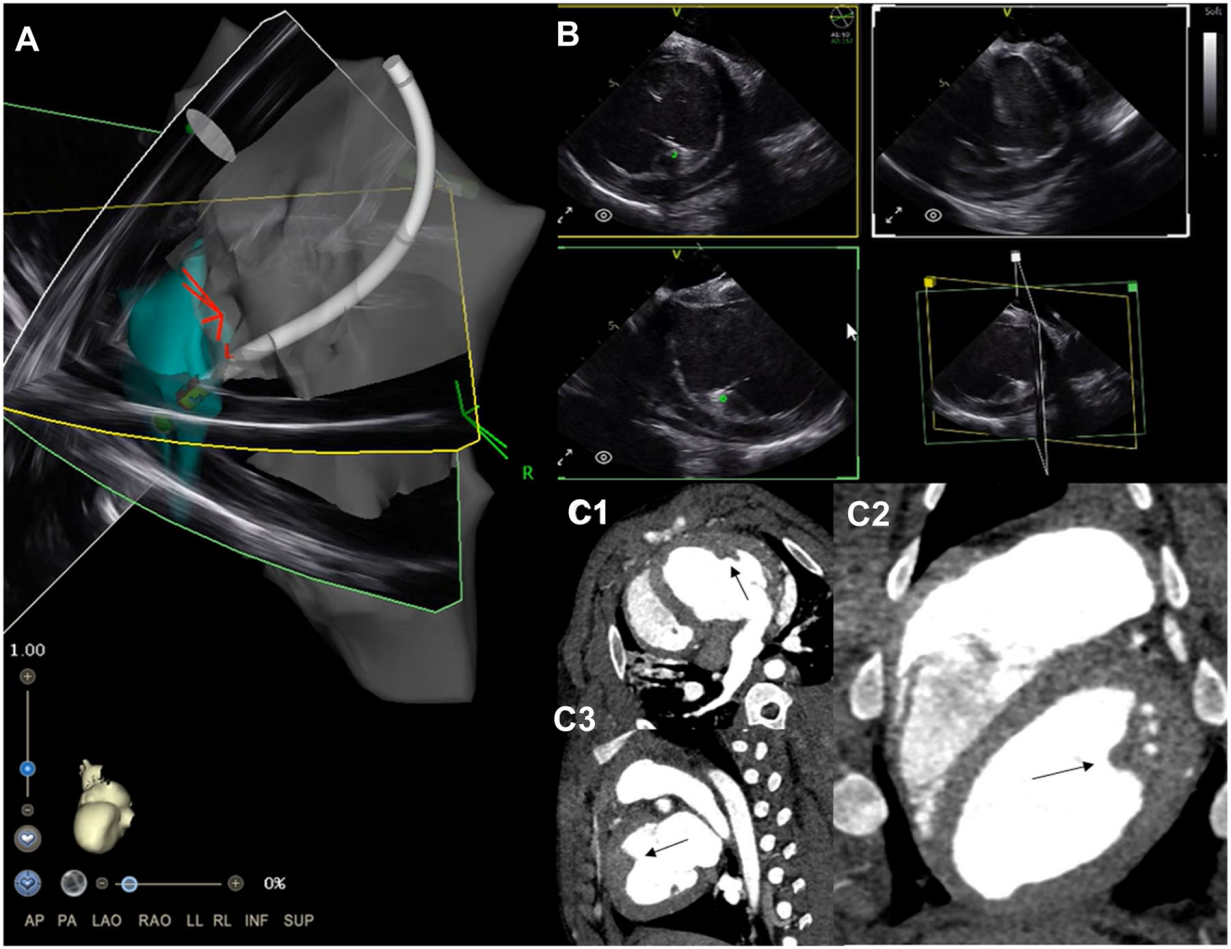
4D ICE guiding ablation of the anterior papillary muscle using Carto (A), multiplane images from the 4D ICE catheter showing localization of the ablation catheter tip (green dot) on the papillary muscle in multiple views (B), and CT images showing the structure of the papillary muscle on CT compared to 4D ICE in the transverse (C1), coronal (C2), and sagital (C3) planes.

**Figure 4:**
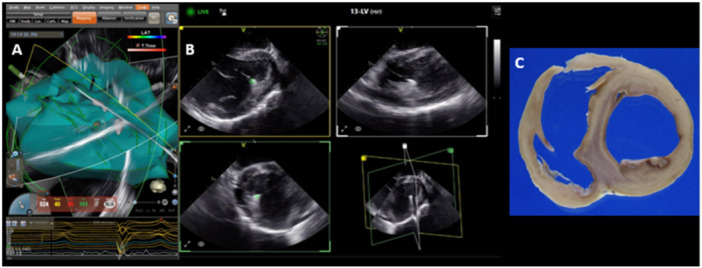
4D ICE guiding ablation of the inferior papillary muscle using Carto (A), multiplane images from 4D ICE catheter showing localization of ablation catheter tip (green dot) on the papillary muscle in multiple views (B), and gross pathology of the ablation lesion (C).

**Figure 5:**
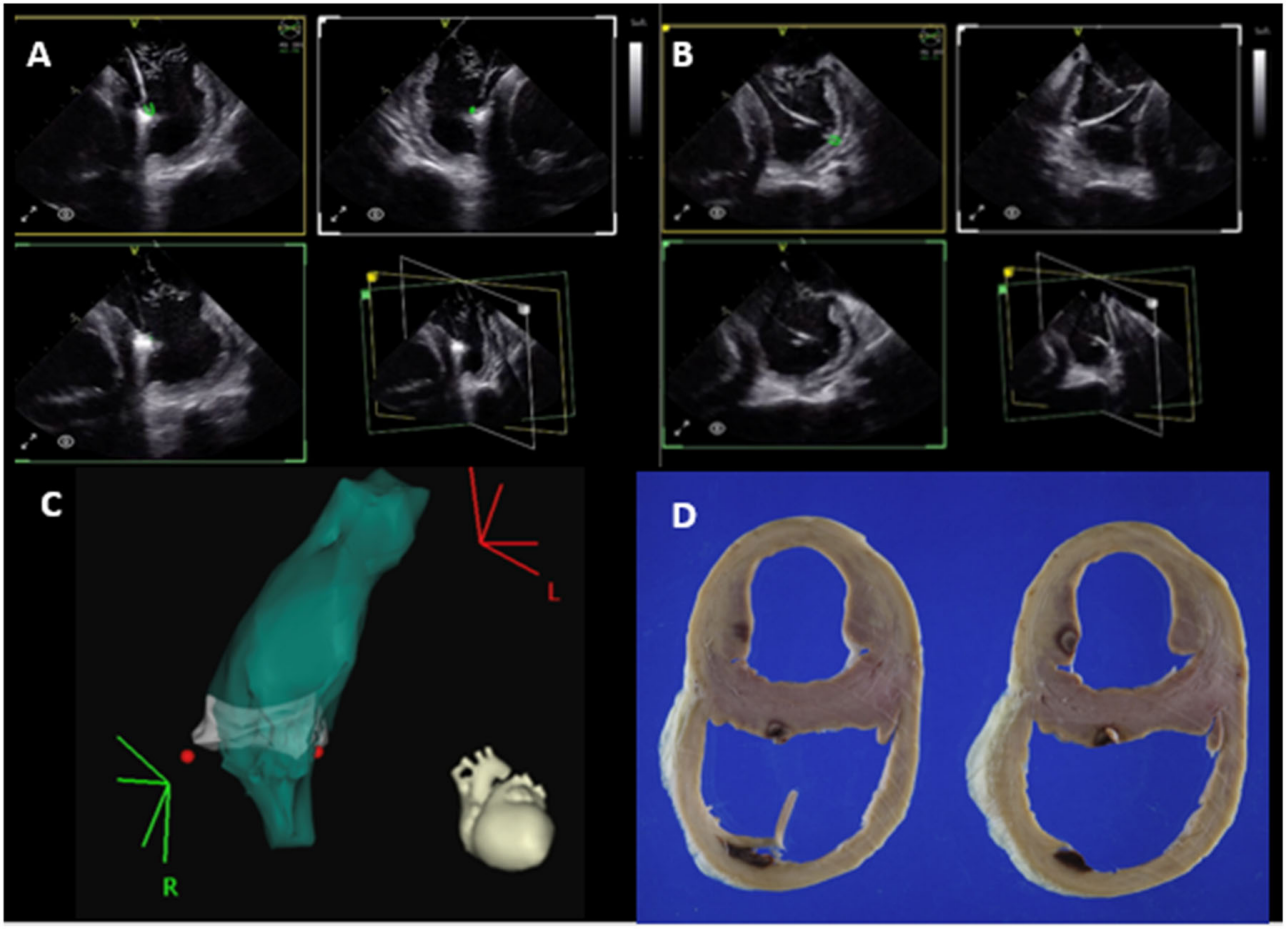
4D ICE guiding ablation of the septal and free wall insertions of the RV moderator band using Carto. Multiplane images from 4D ICE catheter showing localization of ablation catheter tip (green dot) on the septal (A) and free wall (B) of the RV moderator band in multiple views. Carto shell created using CARTOSOUND of RV and RV moderator band (C), and gross pathology of the ablation lesions (D).

**Figure 6:**
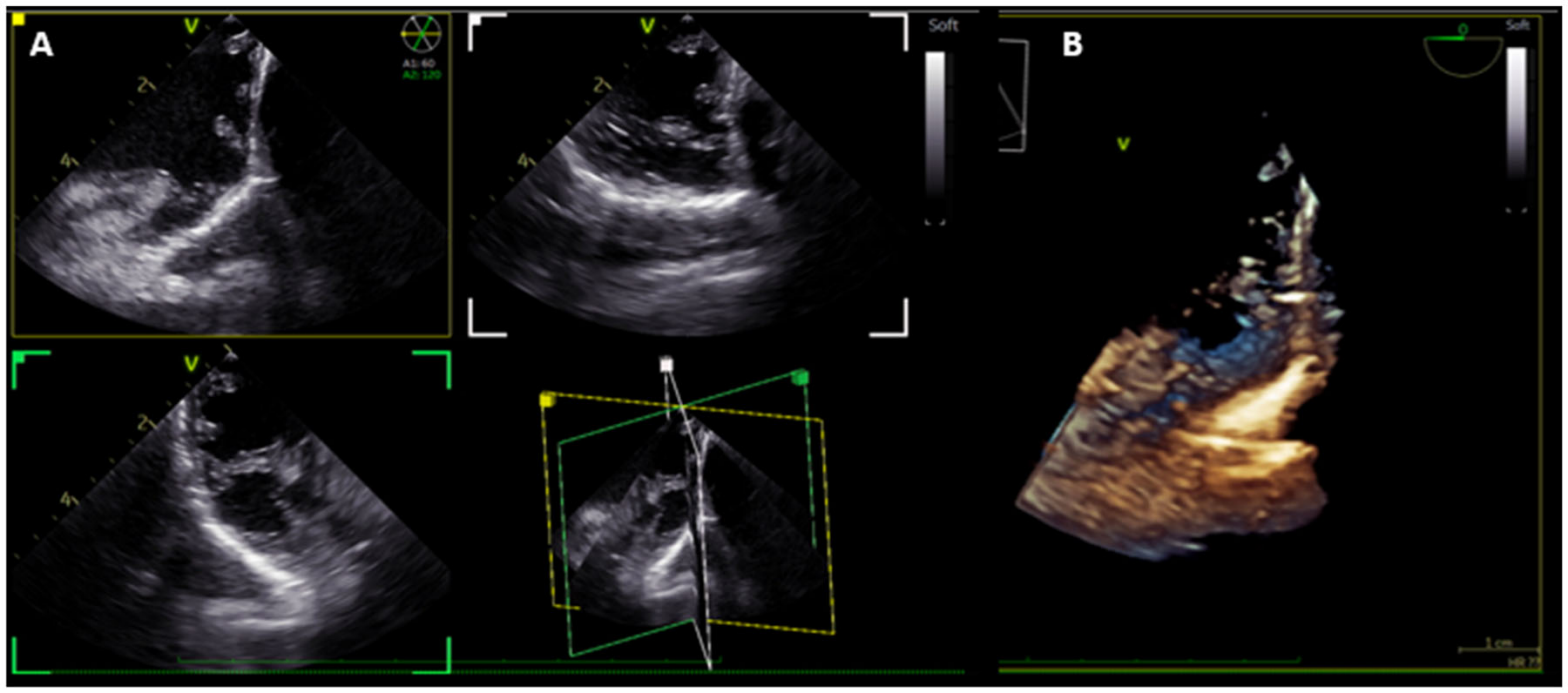
4D ICE imaging of the left atrial appendage showing multiplane images of the LAA (A) and 3D volumetric reconstruction of the LAA (B).

For each subject, the RV, LV, and LA were reconstructed using CARTOSOUND to create a basic map of the anatomy. This process was completed using the novel multiplane imaging software platform, which allowed for visualization of three separate planes simultaneously. Endocardial tracings can be taken with CARTOSOUND in each of the planes allowing for creation of anatomy with minimal movement of the ICE catheter (Figure 1). These slices can also be orthogonal to each other allowing for proper dimensioning of the structure without complex catheter manipulation. Maps generated were similar to 3D reconstruction acquired in pre‐procedure CT scans (Figure 2 and 3). Subsequently, ablation lesions were successfully delivered to the LV papillary muscles (Figure 4) and RV moderator band (Figure 5) with 4D ICE guidance. Multiplane imaging allowed for more accurate placement of the ablation catheter directly on the tip of the papillary muscle rather than on the side, and at the lateral insertion of the moderator band, as it could be visualized in multiple views simultaneously. This can be seen on gross pathology as well, where lesion location was well correlated with location on the electroanatomic map (EAM) and ICE images (Figures 3 and 4). During ablation, one steam pop occurred and was visualized on ICE. After ablation, the left atrium was accessed via a transseptal approach and the 4D ICE catheter was advanced into the left atrium. 2D, multiplane, and 3D volumetric images were obtained of the LAA to simulate use for an LAAO procedure (Figure 6). Specific views were easily obtained with rotation of the imaging plane to obtain measurements for an LAAO device without movement of the catheter.

## 4 Discussion

Procedural guidance has advanced considerably over the years from fluoroscopy to EAM and intracardiac ultrasound (ICE). The use of ICE has allowed for high‐resolution visualization of intracardiac structures in real time, something previously not possible with fluoroscopy and very difficult with EAM. 4D ICE is the natural progression of this technology, adding features previously only seen on TEE probes like omniplane, multiplane imaging, and 3D volumetric imaging. These features allow for improved visualization of cardiac structures for creation of anatomy with CARTOSOUND, ablation of complex intracardiac structures like the papillary muscles and the moderator band, and could even replace TEE for structural procedures like LAAO.

In our preclinical evaluation, in each of the seven swine, ICE was advanced into the RA, RV, and LA and used for CARTOSOUND reconstruction of the left ventricle, papillary muscles, right ventricle, moderator band, and LAA. Use of CARTOSOUND to create a map of basic anatomy can be beneficial over FAM as heart borders are not subject to deformation by the mapping catheter. This comes with the downside of operator‐dependent variability and more frequent, and sometimes complex, catheter manipulation to obtain ideal views and image quality. New deep learning algorithms have decreased operator‐dependent variability, substantially decreased the amount of time to create a reconstruction of the LA, and demonstrated qualitative similarity with CT measurements [9]. With 4D ICE, multiple 2D images can be obtained simultaneously, which could facilitate more rapid delineation of 3D anatomy with less catheter manipulation. In this study, auto‐contouring AI algorithms were not available to directly compare the speed of sound to FAM with manual contouring versus AI auto‐contouring in a qualatative manner, though this has been previously reported [9]. Additionally, time to chamber reconstruction was not directly measured, limiting the quantitative comparison of 2D–4D ICE catheters for the creation of geometry. Obtaining ideal images for reconstruction was more straightforward though, as fine‐tuning and acquiring additional images can often be done by rotating the transducer field of view without physically moving the catheter. This could decease procedural times and potentially decrease complications related to frequent manipulation of the ICE catheter.

Beyond initial creation of anatomy, 4D ICE has a potential benefit to guide ablation of complex intracavitary structures. Ablation of premature ventricular contractions (PVCs) or ventricular tachycardia (VT) from structures like the moderator band and papillary muscles can be challenging as these are multifaceted 3D structures that can have significant movement during the cardiac cycle and a large amount of variability between patients. These structures are difficult to accurately map with mapping catheters alone as FAM is designed to assign points to and interpolate between the outermost locations reached by the catheter. This works well for delineation of the endocardium, but often struggles to define intracardiac structures, which are, by definition, internal to the surrounding points. Since these structures cannot be seen on fluoroscopy, 2D ICE has been revolutionary for real‐time guidance of ablation in these areas [8, 10, 11]. Lesion depth and quality are also directly related to tissue contact, which can be difficult to maintain when additionally considering the small surface area of the structures. With 4D ICE, multiplane live imaging allows for direct real‐time visualization of the ablation catheter and intracavitary structure in three planes. Though this does not necessarily enhance the fidelity of the sound to FAM reconstruction, in this study, it enhanced the ability to position the catheter on elusive structures such as the tip of the papillary muscle or the body of the moderator band. The smaller the surface area of the target location, the more difficult it is to appreciate on 2D imaging as it can move in and out of plane or have a relatively small cross‐sectional area in the plane of imaging most easily obtained. With multiplane imaging, these irregularly shaped structures can be better appreciated with minimal catheter manipulation, which facilitated optimal ablation catheter positioning. This is demonstrated in Figure 4 as the catheter was able to be manipulated to the papillary muscle allowing targeted delivery of a single lesion with relative ease. Additionally, papillary muscle structure was visually similar between the 4D ICE images and corresponding CT images, which can be seen in Figure 3.

Beyond ablation of complex intracavitary structures, 4D ICE also showed promise as a method to visualize the LAA and guide LAAO procedures. During the procedure, the 4D ICE catheter was advanced through a transseptal puncture into the LA. From the LA, multiplane and 3D volumetric images were obtained of the LAA allowing for better delineation of anatomy, similar to TEE, without the need to manipulate the ICE catheter to different positions (e.g., pulmonary vein, etc.) for different views. For structural procedures like LAAO, this allows for accurate measurement of LAA anatomy, sizing of the occlusion device, procedural guidance, and evaluation for peri‐device leaks and complications. As leaks can be eccentric and small, visualization of a Doppler jet for proper diagnosis and grading can be difficult with only a 2D catheter. The 4D ICE catheter allows for visualization of color Doppler in multiple views simultaneously, and more comprehensive 360° coverage of the LAAO device, facilitating identification and measurement of leaks. Additionally, compared to TEE, ICE is well tolerated, can be performed in patients with esophageal pathology, and avoids the need for general anesthesia. It can also potentially simplify procedural logistics as a separate TEE operator is no longer needed, provided the primary operator is experienced with LAAO device placement and ICE imaging, including manipulation of the imaging console. Taken together, this could reduce procedure times and improve patient safety.

Though there are many benefits to 4D ICE, there are some drawbacks. The catheter is larger in diameter than a typical 2D ICE catheter that is compatible with CARTOSOUND, requiring 11Fr venous access instead of the typical 9Fr access for the 8Fr catheter. This may increase vascular complications and bleeding. Additionally, imaging of the LAA was attempted from the RA and RV outflow tract, but the LAA could not be completely visualized. Though it might be possible to visualize the LAA from the pulmonary artery, which was not attempted in this study, procedural guidance will likely require placement of the ICE catheter in the LA. This would require a second transseptal puncture and the associated risk, or expansion of an already large first transseptal to accommodate the size of both the ICE catheter and LAAO device delivery system.

As with most technology, the increased complexity comes with increased cost, which requires careful consideration to ensure the additional functionality is warranted for a given procedure. The increased complexity of 3D image processing also currently requires an additional technician, or even a dedicated imager, in the EP lab to operate the ultrasound machine, unlike the more streamlined workflow of 2D ICE. For many ablations, including pulmonary vein isolation, typical and atypical atrial flutter, outflow tract PVCs, and many supraventricular tachycardias, it is unlikely that 4D imaging adds much to procedural navigation and success. These procedures often don't involve complicated intracavitary structures and therefore can be easily guided by a combination of EAM, 2D ICE, and limited fluoroscopy. That being said, it is possible that future software enhancements (e.g., AI‐driven sound to FAM for all cardiac chambers) could reduce procedure time and fluoroscopy exposure, strengthening the argument for 4D ICE in all ablation cases.

## 5 Limitations

This was a preclinical study in a small number of animals performed to assess the function and potential clinical utility of 4D ICE for guiding invasive electrophysiology procedures. It is therefore limited by the cohort size as well as the ability to directly compare the experiences in porcine and humans. A comprehensive quantatative analysis of the speed of sound to FAM reconstruction as well as a side‐by‐side comparison to TEE was not performed. Additional investigation is required to further determine the clinical value of this emerging technology in patients.

## 6 Conclusion

Intracardiac echocardiography (ICE) is an essential imaging modality for electrophysiology procedures, allowing intraprocedural monitoring, real‐time catheter manipulation guidance, and visualization of complex anatomic structures. 4D ICE is the next stage in evolution of the technology, permitting 360° rotation of the imaging plane, simultaneous multiplanar imaging, and volumetric acquisition, similar to TEE. Integration within the electroanatomical mapping system software platform may provide additional value for guiding ablation of challenging intracavitary structures and is a novel feature of the NuVision catheter. Though promising, this technology is new and further investigation is warranted to determine its clinical value during electrophysiology and structural heart procedures.

## Conflicts of Interest

Dr. Tschabrunn receives grant support from Biosense Webster, Abbott Medical, Baylis Medical, CIRCA Scientific, and the National Institutes of Health and honoraria for consulting and lecturing from Abbott Medical and Baylis Medical. Dr. Callans receives honoraria for consulting and lecturing from Abbott Medical, Biosense Webster and Boston Scientific. Dr. Brem is an employee of Biosense Webster. The other authors declare no conflicts of interest.

## Data Availability

The data that support the findings of this study are available from the corresponding author upon reasonable request.

## References

[1] T. Bartel , S. Muller , A. Biviano , and R. T. Hahn , “Why Is Intracardiac Echocardiography Helpful? Benefits, Costs, and How to Learn,” European Heart Journal 35, no. 2 (2014): 69–76, 10.1093/eurheartj/eht411. 24144789 PMC3882724

[2] M. Hanson and A. Enriquez , “Intracardiac Echocardiography to Guide Catheter Ablation of Idiopathic Ventricular Arrythmias,” Cardiac Electrophysiology Clinics 13, no. 2 (2021): 325–335, 10.1016/j.ccep.2021.03.010. 33990271

[3] P. C. Qian and U. B. Tedrow , “Intracardiac Echocardiography to Guide Catheter Ablation of Ventricular Arrhythmias in Ischemic Cardiomyopathy,” Cardiac Electrophysiology Clinics 13, no. 2 (2021): 285–292, 10.1016/j.ccep.2021.02.002. 33990267

[4] M. E. Field , M. R. Gold , M. R. Reynolds , et al., “Real‐World Outcomes of Ventricular Tachycardia Catheter Ablation With Versus Without Intracardiac Echocardiography,” Journal of Cardiovascular Electrophysiology 31, no. 2 (2020): 417–422, 10.1111/jce.14324. 31868258

[5] A. Jhand , A. Thandra , Y. Gwon , et al., “Intracardiac Echocardiography versus Transesophageal Echocardiography for Left Atrial Appendage Closure: An Updated Meta‐Analysis and Systematic Review,” American Journal of Cardiovascular Disease 10, no. 5 (2020): 538–547. 33489456 PMC7811919

[6] A. Patel and M. Valderrábano , “Role of Intracardiac Echography for Transcatheter Occlusion of Left Atrial Appendage,” Cardiac Electrophysiology Clinics 13, no. 2 (2021): 313–323, 10.1016/j.ccep.2021.02.004. 33990270

[7] M. Alkhouli , Z. M. Hijazi , D. R. Holmes , C. S. Rihal , and S. E. Wiegers , “Intracardiac Echocardiography in Structural Heart Disease Interventions,” JACC: Cardiovascular Interventions 11, no. 21 (2018): 2133–2147, 10.1016/j.jcin.2018.06.056. 30409271

[8] A. Enriquez , L. C. Saenz , R. Rosso , et al., “Use of Intracardiac Echocardiography in Interventional Cardiology Working With the Anatomy Rather Than Fighting It,” Circulation 137, no. 21 (2018): 2278–2294, 10.1161/CIRCULATIONAHA.117.031343. 29784681

[9] L. Di Biase , F. Zou , A. N. Lin , et al., “Feasibility of Three‐Dimensional Artificial Intelligence Algorithm Integration With Intracardiac Echocardiography for Left Atrial Imaging during Atrial Fibrillation Catheter Ablation,” EP Europace 25, no. 9 (2023): euad211. 10.1093/europace/euad211 PMC10403247 37477946

[10] A. Enriquez , C. Tapias , D. Rodriguez , et al., “Role of Intracardiac Echocardiography for Guiding Ablation of Tricuspid Valve Arrhythmias,” HeartRhythm Case Reports 4, no. 6 (2018): 209–213, 10.1016/j.hrcr.2018.02.010. 29922578 PMC6006487

[11] A. N. Lin , Y. Shirai , J. J. Liang , et al., “Strategies for Catheter Ablation of Left Ventricular Papillary Muscle Arrhythmias: an Institutional Experience,” JACC. Clinical Electrophysiology 6, no. 11 (2020): 1381–1392. 33121667 10.1016/j.jacep.2020.06.026

